# Cardio-rheumatology: the cardiovascular, pharmacological, and surgical risks associated with rheumatological diseases in women

**DOI:** 10.1139/cjpp-2023-0420

**Published:** 2024-03-15

**Authors:** Samantha Le Sommer, Maria I. Kontaridis

**Affiliations:** a Department of Biomedical Research and Translational Medicine, Masonic Medical Research Institute, Utica, NY, USA; b Department of Medicine, Division of Cardiology, Beth Israel Deaconess Medical Center, Boston, MA, USA; c Department of Biological Chemistry and Molecular Pharmacology, Harvard Medical School, Boston, MA, USA

**Keywords:** cardio-rheumatology, women’s health, cardiovascular disease, rheumatic diseases, lupus, autoimmunity

## Abstract

Cardiovascular disease (CVD) remains the number one cause of death worldwide. Women are at increased risk of death from CVD, but the mechanisms for how and why this occurs remain elusive. One subset of women who are exceptionally vulnerable to CVD are those with rheumatic diseases (RDs). Indeed, women account for 80% of all RDs, disorders that encompass a broad range of autoimmune and autoinflammatory diseases that lead to chronic inflammation and pathology. The clear association of increased CVD risk in women with RD is thought to be mediated by a number of factors, including RD pathology itself, pharmacological induction of CVD, and/or as yet unidentified mechanisms. As such, elucidation of the causes and treatments of these pathologies has given rise to a new subspecialty of cardiology: cardio-rheumatology. Here, we review and discuss the CVD risks in patients with RDs, the associated sex disparities in RD and CVD care, as well as the current therapeutic and interventional options available to specifically help women with RDs. We hope this discussion will provide guidance and support to patients, as well as to cardio-rheumatologists, as these groups are the most uniquely positioned to radically improve CVD care in these individuals. Moreover, we are hopeful this discussion may lead to better, more efficacious approaches to treating these disorders in women in the near future.

## Introduction

Cardiovascular diseases (CVDs), which include coronary heart disease, stroke, high blood pressure, heart failure, and diseases of the arteries, are responsible for approximately 1 million deaths per year in the US alone (Tsao et al. 2023). Despite the tremendous efforts within the field over the last several decades, including the development and use of blood-pressure lowering medications, statins, and the invention of life-saving devices such as the pacemaker and defibrillator (Willy et al. 2022), the incidence and mortality of CVD is and remains the number one cause of death worldwide. Moreover, and perhaps more concerningly, any improvements and successes in CVD diagnosis and treatment have not been shared equally amongst the sexes; until recently, most CVD research had only been conducted in men or in male mice. Even the most recent CVD clinical trials are still comprised of ~65% men (Tobb et al. 2022), and further, women who do partake in such trials tend to be post-menopausal. As such, we still know little about the sex-specific mechanisms of CVD development in women.

Generally, premenopausal women have a lower risk of CVD than age-matched men, presumably because of protective effects of higher estrogen. However, the risk of CVD in this group increases significantly when there is comorbidity with a rheumatic disease (RD) (Mehta et al. 2023). RDs display significant sex bias towards women, where 80% of individuals diagnosed are women (Angum et al. 2020). Therefore, despite most women being protected from CVD before the age of 55, this effect is lost in individuals with RDs and the risk for CVD becomes equally prevalent between men and women, even at this younger age (Moran et al. 2022).

**Fig. 1.**
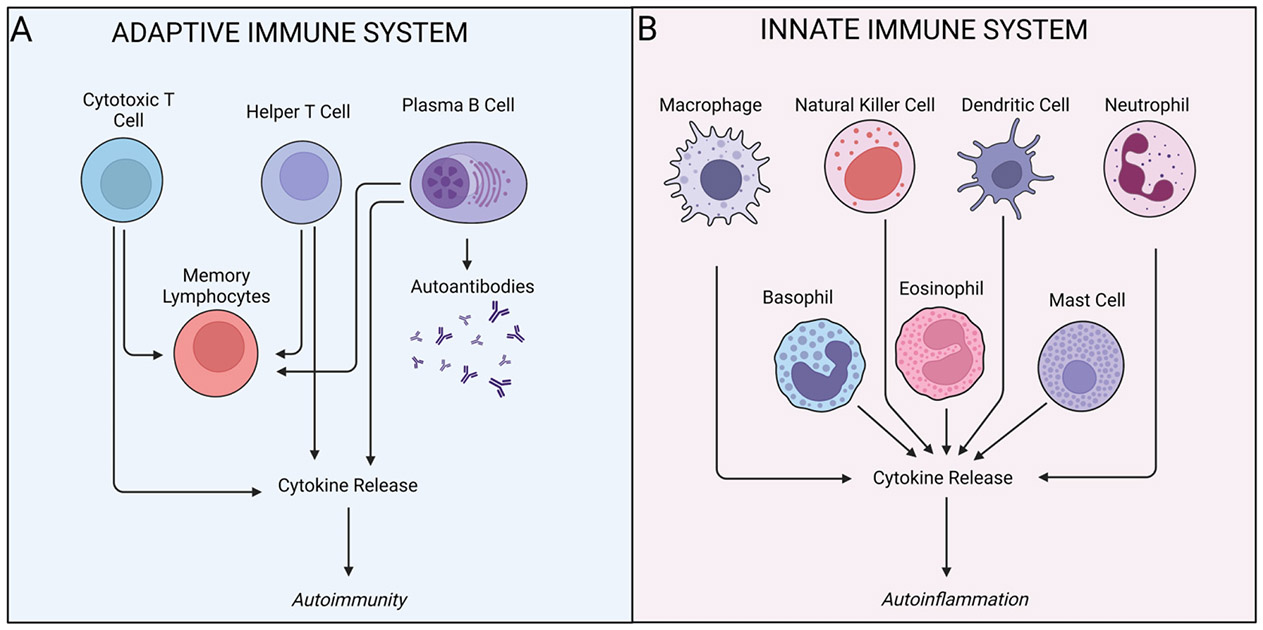
Autoimmunity is driven by the adaptive immune system, while autoinflammation is innate driven. (A) Autoimmune diseases like rheumatoid arthritis result in chronic inflammation mediated by antigen specific B and T cell immune responses. Self-reactive cytotoxic T cells and helper T cells produce pathogenic cytokines which create and reinforce the inflammatory milieu. Meanwhile, activated plasma B cells produce autoantibodies, which further drive antigen specific attack. (B) Autoinflammatory diseases, on the other hand, are mediated by the innate immune system, which consists of a number of cells such as macrophage, natural killer cells, dendritic cells, neutrophils, basophils, eosinophils and mast cells, all of which recognize broad categories of molecular pattern receptors that facilitate responses to polysaccharides and nucleic acids. These innate immune cells produce a range of pro-inflammatory cytokines which ultimately lead to autoinflammation and pathology.

RDs are a diverse group of autoinflammatory/autoimmune conditions that affect the joints, tendons, muscle, ligaments, bones, and muscle (Sangha 2000). Approximately 10% of the global population is diagnosed with an autoimmune disorder, making this one of the most prevalent chronic classes of disorders worldwide (Miller 2023). In autoimmune diseases, the adaptive immune system initiates a B and T cell driven antigen-specific immune response (Fig. 1A) (Theofilopoulos et al. 2017) targeting, for example, nuclear proteins in systemic lupus erythematosus (SLE) (Riemekasten and Hahn 2005) or homocitrulline and citrulline in rheumatoid arthritis (RA) (Shi et al. 2011; Scinocca et al. 2014; Turunen et al. 2015). Autoinflammatory disorders, by contrast, are a relatively new, but similar, group of diseases, whereby the innate immune system initiates a non-antigen specific immune response directed against the host (Fig. 1B) (Krainer et al. 2020). Unlike the classic autoimmune diseases, autoinflammatory disorders are driven by macrophages, neutrophils, and dendritic cells, and can result in either systemic or site-specific inflammatory reactions (Havnaer and Han 2019). One example for this class of disorders is gout, an autoinflammatory disease that leads to joint inflammation (Galozzi et al. 2021).

Importantly, RDs in women increase CVD risk and require a need for specialized care. A myriad of factors play a role in the complexities of CVD comorbidities in RD patients, including systemic inflammation, cardiotoxicity associated with RD drug treatments, and the atypical presentation of CVD in these patients (Alhusain and Bruce 2013). These effects, therefore, have recently given rise to a new subspecialty of cardiology: cardio-rheumatology. Here we discuss the CVD risks in patients with RDs, the associated sex disparities in RD and CVD care, as well the current therapeutic and interventional options available to specifically help women with RDs. We hope this discussion will provide guidance and support to patients, as well as to cardio-rheumatologists, as these groups are the most uniquely positioned to radically improve CVD care in these individuals (Prasad et al. 2014). Moreover, we are hopeful this discussion may lead to better, more efficacious approaches to treating these disorders in women in the near future.

## RDs and CVDs have overt sex biases

RDs have a striking imbalance between sexes, disproportionately affecting women over men (Table 1) (Voskuhl 2016). This is especially apparent in autoimmune disorders such as Hashimoto’s (19:1) (Calcaterra et al. 2020); SLE (9:1) (Rider et al. 2018), RA (3:1) (Maranini et al. 2022), and antiphospholipid syndrome (3.5:1) (Angum et al. 2020), where the female: male ratios are profoundly skewed (Table 1). Despite these stark observations, the precise functional mechanisms causal to these sex differences in RDs remains unclear.

Conversely, sexual dimorphism in the immune system has been well-documented. Women have a lower threshold for immune activation (Gal-Oz et al. 2019), leading to increased inflammatory responses compared with men. It has long been speculated that despite its potential cardioprotective functions, estrogen may increase the risk of developing RDs (Desai and Brinton 2019). In this regard, there are new emerging cases of RDs stemming from individuals undergoing estrogen gender-affirming hormone therapy (White et al. 2022). However, to date, no direct evidence that high levels of estrogen are causal to RD has been found (Rider et al. 2018; Desai and Brinton 2019; Kim et al. 2022).

Despite this, hormones have been long associated with progression of RD pathogenesis, as both estrogen and testosterone elicit responses from immune cells. Specifically, estrogen protects autoreactive B cells from negative selection leading to decreased self-tolerance. In autoimmune disorders such as SLE, both high dose estrogen therapy and pregnancy (where estrogen levels increase) correlate with worsened disease activity. In men with SLE, reduced testosterone is similarly correlated with increased disease burden, suggesting that the imbalance of hormones may be prognostic to progression and severity of RDs.

Though biological basis for sex disparities in RDs exists, it is important to note that sex imbalance does not exist in isolation, and it can be made more complicated by the already existing biases for women in medicine and clinical practice (Emambokus et al. 2021, 2022). Therefore, it is difficult to detangle the effects of sex determinants in diagnosis, treatment, and/or outcomes for women who concomitantly have CVD and RD (Burgess 2022). Even so, multiple meta-analyses studies have shown that medical professionals are more likely to dismiss female patients, in general, as they are considered to be too sensitive, too emotional, too wordy, and time wasters (Samulowitz et al. 2018). This is especially true for chronic conditions like RDs that cause pain, where women are more likely to underestimate their degree of tolerance on an arbitrary threshold scale. Irrespective of the reasoning, it is clear that women generally tend to be prescribed less medication, more antidepressants, and get increased numbers of referrals for mental health services than men (Weisse et al. 2021). More needs to be done to help women get proper diagnosis and more effective care on this front.

It is noteworthy that women have a 2-fold increased mortality over men following myocardial infarction (MI). With respect to women with RDs, this number is even more astounding, as these patients have a 15% even greater risk of getting an MI (Wassif et al. 2022). This may be mediated, at least in part, by the fact that some primary care physicians believe women with RDs are more protected against development of CVDs due to their having elevated estrogen levels, leading to less aggressive and/or later management of care for CVD symptoms in these individuals (Desai et al. 2021). Consequently, numerous studies and analyses now stress the urgency in increasing training amongst health care professionals, to better understand and recognize sex- and gender-specific CVD symptoms, risk, presentation, and management, especially amongst the RD group (Mosca et al. 2011; Norris et al. 2020; Adreak et al. 2021; Wenger et al. 2022). Sadly, despite new regulations and programs to reduce gender disparities, there are, to date, no established protocols for managing risks of CVD in women with RDs.

However, there are additional considerations affecting women’s CVD prognosis. These include the fact that women are less likely to accurately assess their own CVD symptoms and women often take longer to call emergency services when experiencing an MI than men (Weininger et al. 2022). This misinterpretation of MI symptoms can lead to, on average, a 2 h delay in seeking care (Bugiardini et al. 2017; Stehli et al. 2021), a critical window that can mean either life or death for these individuals. Worse, even after arriving at a care facility, women with an ST-elevation MI typically have at least a 30 min longer ischemic wait time than men (Bugiardini et al. 2017). Collectively, these data demonstrate the need for increased public awareness of CVD in women, increased need for training in assessing CVD risk in RD patients in both primary and emergency care, and improved guidelines on appropriate referral pathways for RD patients that include guidance from cardio-rheumatologists directly.

## RD leads to an increase in CVD risk

Multiple biological factors have also been suggested to contribute to the increased risk of CVD in RDs, although studies so far tend to be contradictory. For example, both too much and too little inflammation has been implicated as causal to increased CVD risk (Tall and Fuster 2022). In RD, chronic inflammation that is characteristic of RDs is considered to accentuate already existing CVD risk factors (Lazou et al. 2020). Moreover, RDs, in and of themselves, may also directly mediate cardiac pathophysiology (Table 2). The strongest evidence for this comes from a large population study of RA, where even after correction of socioeconomic, education, and income factors, patients demonstrated an unexplained 50% increase in CVD risk (Maradit-Kremers et al. 2005; Solomon et al. 2006). More recently, a population study by Conrad et al. examined the electronic medical records of 22 million individuals in the United Kingdom and found that there was an increased risk for CVD in all 19 autoimmune diseases examined by the study (Conrad et al. 2023). Interestingly, the risk was progressive and compounded by the number of autoimmune diseases present in any given individual (Conrad et al. 2023). These studies highlight the urgent need for increased surveillance of CVD in individuals with RDs.

Lifestyle factors can also affect CVD risk in RD, as the diagnosis itself can make an active and healthy lifestyle difficult to maintain and/or achieve due to decreased mobility, fatigue, and chronic pain (Overman et al. 2017). Likely, it is the combination, and not one single factor described above, that mediates the increased CVD burden and mortality in individuals with RDs.

**Fig. 2.**
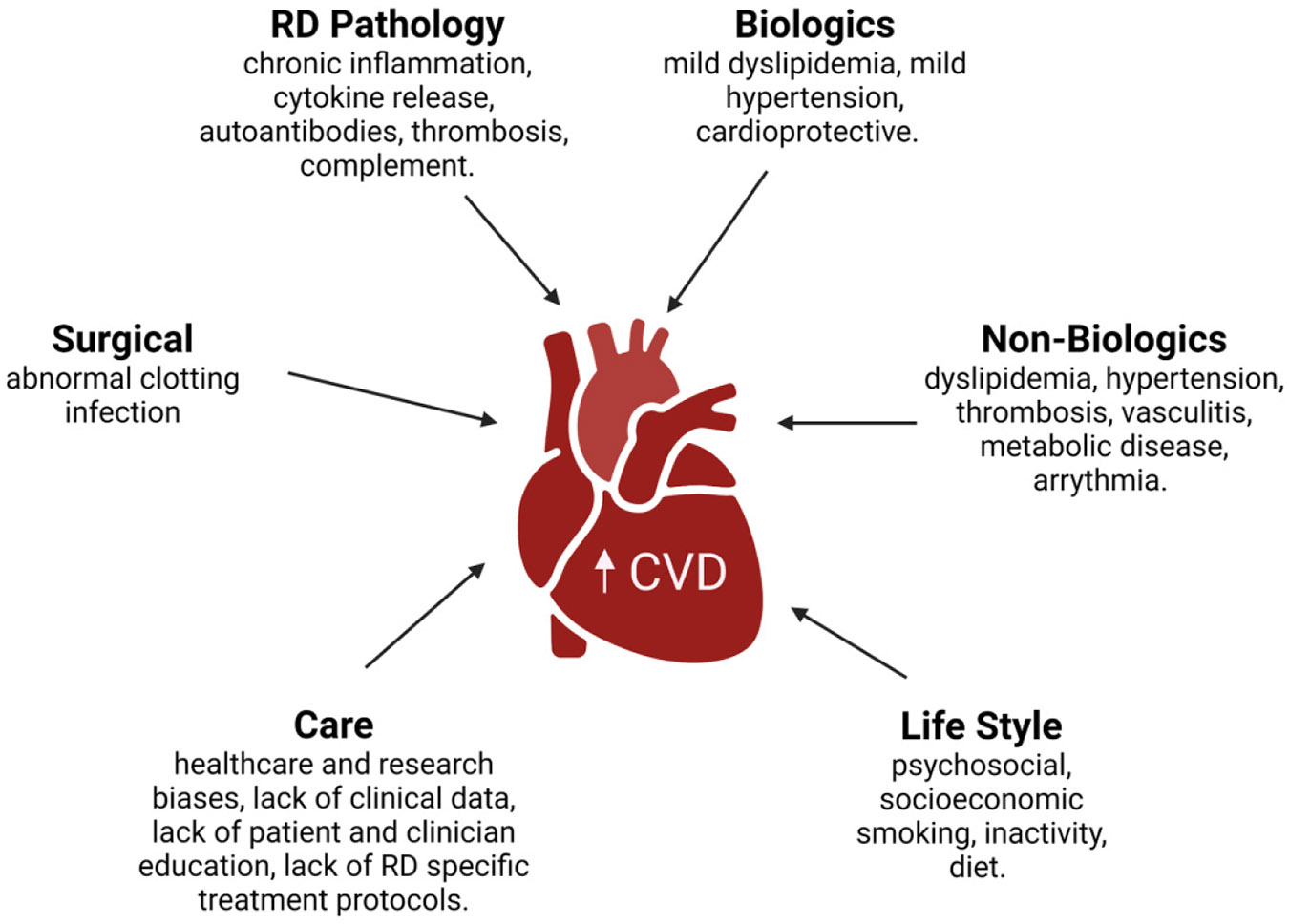
There are multiple factors that contribute to increased cardiovascular disease (CVD) burden in women with rheumatic diseases (RDs). RD pathology itself, therapeutics in the form of biological drugs and non-biological drugs, lifestyle, standard of care, and increased risk of complications following surgery, all act in concert to increase CVD risk and mortality in women with RDs.

To add to the complexity, CVD in women with RDs can present with atypical or misleading phenotypes (Fig. 2). Paradoxical presentations in RDs include the fact that many have decreased levels of LDL cholesterol; unlike non-RD individuals, RD patients with low LDL have increased CVD risk and mortality (Elena et al. 2011; Behl et al. 2020; Robinson et al. 2022). Furthermore, women under 65 are more likely than men to suffer an MI without the classic sudden chest pain, often reporting much milder symptoms and discomfort in the back, arm, or neck. More work in parsing out the clinical relationship between RDs and early clinical warning signs of CVD is required to improve early diagnosis and subsequent long-term outcomes.

Taken together, it is clear that CVD risk and associated complications in women are mediated by several mechanisms acting in concert, including factors that influence biological, social, genetic, and lifestyle risk, as well as tangential mediators that can lead to unrecognized, delayed, less aggressive, or misdiagnosed incidences of CVD, particularly in RD patients.

## Non-biologic pharmacological management of RD and CVD risk

Pharmacological management of RDs, especially in the context of CVD, can be complex (Table 3). Corticosteroids, a commonly used class of drugs that can ameliorate symptoms without improving RD pathology, have long been recognized as causal to cardiovascular side effects (Pujades-Rodriguez et al. 2020). In the short term, some studies have found their use beneficial (Makheja et al. 1989; Hagihara et al. 1991); however, long term treatment with corticosteroids can lead to dyslipidemia, hypertension, systemic vascular resistance, increased extracellular volume, increased cardiac contractility, left ventricular wall rupture and delayed myocardial scar formation post MI (Kelly et al. 1998; Schäcke et al. 2002, Hafezi-Moghadam et al. 2002). In addition, corticosteroids have also more recently been implicated in altering lipid metabolism, leading to accumulation of lipids, pro-atherogenic dyslipidemia, and endothelial injury (Arnaldi et al. 2010; Gökalp et al. 2017). However, the functional molecular mechanisms leading to the development of these corticosteroid-associated deleterious side effects have yet to be fully elucidated.

No direct studies on sex-specific responses to corticosteroids in RDs have been conducted. However, it may be possible that this class of drugs can further existing CVD risk factors in patients with RDs. While low dose or alternate day dosing regimens for corticosteroids have been suggested to lower risks, for many RD patients, these drugs are considered a salvation, and dosing can be difficult to manage or alter (Chaia-Semerena et al. 2020). Clearly, RD treatment drugs need to be finely balanced between resolving RD symptoms and protecting the cardiovascular system.

Methotrexate is another first-line immunosuppressive drug option for RDs (Hamed et al. 2022). Unlike corticosteroids, methotrexate is considered to be cardioprotective (De Vecchis and Palmisani 2016; Mangoni et al. 2017a; Bălănescu et al. 2019); RA patients taking methotrexate have a 24% decreased risk of CVD and a 57% reduced risk of hospitalization due to heart failure (Tate et al. 2021). A valuable predictor of CVD is blood pressure, and multiple studies have identified that RDs such as RA result in increased systolic and diastolic blood pressure. (Fuchs and Whelton 2020). It has been demonstrated that methotrexate lowers both peripheral systolic and diastolic blood pressure in RA patients, and results in reduced arterial stiffness as assessed by pulse wave velocity, regardless of sex (Mangoni et al. 2017b). Interestingly these studies all conclude that, once stratified for sex, methotrexate treatment equally reduces risk of hypertension and CVD in both males and females (Mangoni et al. 2017b; Kim et al. 2020; Tate et al. 2021). However, a comparison of sex response to drug response was not the primary goal of these studies, and several were either conducted only in men or were insufficiently powered to make these conclusions. Therefore, to definitively prove methotrexate is equally protective in both men and women, additional comprehensive and functional assessments need to be demonstrated, ones that are specifically designed to examine sex differences as the major biological readout. Irrespective, and despite the seemingly significant improvements in hypertension and CVD risk, methotrexate is teratogenic (Mantilla-Rivas et al. 2020). Therefore, its use in younger women remains limited regardless.

Azathioprine is yet another commonly used immunosuppressant in RD that exerts its effects by decreasing nucleic acid synthesis (Broen and van Laar 2020). Though this drug is non-teratogenic, its use has become increasingly less common in recent years due to its newly found carcinogenic properties (Natekar et al. 2011). Concerningly, the cancerous side effects of azathioprine took decades to appear in patients that had been taking this drug for years. Cardiac side effects were also noted, including hypotension, tachycardia, paroxysmal arial fibrillation, and sporadic cases of volumetric shock (Brown et al. 1997; Vianello et al. 2011; Dooremont et al. 2013; Dogan et al. 2015). However, the causal mechanism for the onset of CVD in these patients has not been specifically determined (Pasternak et al. 2013).

Patients with RDs also take non-steroidal antiinflammatory drugs (NSAIDs), which are readily available both over-the-counter and by prescription, and include ibuprofen, naproxen, and diclofenac (Conaghan 2012). While they are best known for their side effects on the gastro-intestinal system, cardiovascular side effects are also common and include increases in blood pressure, thrombotic events, MI, and/or heart failure (Graham et al. 2005; Pepine and Gurbel 2017; Varga et al. 2017). NSAIDs exert their anti-inflammatory effects through inhibition of cyclooxygenase (COX) enzymes. Selectivity within the NSAID class of drugs varies from isoform specific inhibition of COX1 or COX-2, to inhibition of both isoforms. COX selectivity is a factor in NSAID management, and it is known that COX-1 inhibitors are safer in terms of CVD risk but carry considerable gastrointestinal side effects including peptic-ulcers and bleeding (Conaghan 2012). However, COX1/2 inhibitors and COX-2 inhibitors carry considerable cardiotoxicity concerns (Kimmel et al. 2005; Lenzer 2005; Mason et al. 2006). This cardiotoxicity risk is best illustrated by the use of rofecoxib, a COX-2 inhibitor that was initially authorized for treatment of pain in RDs, but that had to be withdrawn due to causing increased incidence of MI in patient-treated cohorts.

In line with these observations, a cardiovascular safety risk assessment from the UK evaluated the side effects of seven NSAIDs (naproxen, ibuprofen, diclofenac, celecoxib, etoricoxib, rofecoxib, and lumiracoxib) and found that all but one, naproxen, led to increased risk for MI, stroke, and death (Sven et al. 2011). Moreover, though naproxen showed the lowest risk for CVD, it was found that it could induce gastrointestinal toxicity, thereby often requiring concomitant proton pump inhibitor therapy to prevent adverse side effects (Tai and McAlindon 2021). The conundrum is that proton pump inhibitors also increase the risk of MI, as well as of renal failure and dementia (Ariel and Cooke 2019). Therefore, it is unclear if the cardiovascular risk benefits of naproxen outweigh the potential need for additional drugs that can induce even more adverse side effects. Further, how NSAIDs impact patients with RDs directly in this regard remains to be fully determined.

Another class of RD drugs are the Janus kinase inhibitors (JKI), in which six different drugs are market approved or in clinical trials for use in RD. All drugs in this class have proven to be effective at treating refractory RDs but are causal to deleterious adverse effects. First generation JKIs, which include ruxolitnib, were utilized for the treatment of immune malignancies. However, during clinical trials, ruxolitnib was found to be cardiotoxic, and its long-term use was associated with increased risk of developing heart failure (Verstovsek et al. 2017). Other first generation JKIs also showed cardiotoxic effects; for instance, a large-scale post-marketing safety study found tofacitinib, used for the treatment of RA, increased risk of sudden cardiac death, as well as incidence of MI, stroke, and thrombosis (Ytterberg et al. 2022). Similarly high-risk RA subpopulations also treated with tofacitinib showed increased risk of cardiovascular side effects, as well as increased incidence for development of melanoma (Fleischmann et al. 2023; Wei et al. 2023). Additional Janus kinase (JAK) inhibitors in this class licensed for use in RDs are baricitinib and upadacitinib. However, while these inhibitors had lower risks for development of MI, both significantly increased the risk of stroke and venous thromboembolism (Genovese et al. 2020; Gerd et al. 2023).

Because of these significant adverse effects, next generation JKIs, including decernotinib, filgotinib, and peficitinib, aimed to have an improved safety profile. Indeed, these inhibitors are not associated with incidence of cardiovascular disease in young or in low CVD risk patients with RD. However, patients with high CVD risk developed hyperlipidemia when treated with decernotinib, and thromboembolism when treated with filgotinib (Mori and Tsunoda 2021) In addition, a 2-year post licensing safety study on the use of peficitinib, another next generation JKI currently only licensed in Japan, found that these patients showed increased risk for both cerebral and lacunar infarctions (Takeuchi et al. 2021).

Though the incidence in CVD and mortality are both increased by use of JKIs, the severity of these outcomes appears variable, and the mechanisms for how they occur remain unclear. It has been speculated that the development of these adverse effects is mediated by the differing selectivity of each of the JKIs (Table 3), though data to support a direct link between JAK selectivity and cardio/cardiovascular toxicity is lacking (Kotyla et al. 2020). Regardless, based on the clinical data, it is clear that use of JKIs in patients with RDs must be carefully considered, and given these outcomes, restricted to patients with low CVD risk and/or only in refractory or nonresponse RDs (Song et al. 2023). In this regard, the FDA now requires a black box warning on the use of tofacitinib, baricitinib, and upadacitinib, consequent to the increased CVD risk (FDA 2021). Moreover, the European Medicines Agency provides guidelines that restrict use of tofacitinib, baricitinib, upadacitinib, abrocitinib, and filgotinib, allowing them only to be used after all other therapeutic options have been exhausted (EMA 2022).

Taken together, non-biologic therapeutics, though useful in treating RDs, have demonstratively increased incidence of adverse effects. Moreover, current non-biologics only relieve symptoms of RD; they do not function to treat the underlying cause of the disease. Further, use of these limited option therapeutics for the relief of RD symptoms appears to come at a cost, increasing RD patient risk for future development of CVD. Clearly, more efficacious and better targeted therapies for treatment of RDs, ones that limit and/or even reduce off-target risks for CVD are urgently needed.

## Biologics for the treatment of RD and CVD Risk

As a step towards this direction, novel therapeutics specifically targeting inflammatory mediators such as anti-TNF*α* and anti-IL-1 have recently become available to RD patients. Importantly, while these drugs were originally developed for RD, applications for these biologics in cardiac disease have recently been considered due to their positive effects on reducing CVD in RD patients (Van Taunay et al. 2018). Specifically, anti-IL-1 and anti-IL-6 biologics approved for RA are now being used to treat large-vessel vasculitis (Paroli 2012). Large scale trials have also demonstrated that anti-IL-1*β* biologics reduce CVD events in patients with prior MI and decrease C-reactive protein by 15% (Abbate et al. 2020). Similar improvements have been demonstrated in patients with RA, psoriatic arthritis and ankylosing spondylitis patients undergoing active treatment with anti-TNF*α* biologics as well. However, the protective effects of anti-TNF*α* therapy appears to be only temporary, as symptoms reappear upon cessation of usage (Bavry et al. 2014). Irrespective, the benefits for treatment with anti-TNF*α*, anti-IL1, and anti-IL-6 therapeutics may have broader applications in the future management of CVD, both for patients with or without RD, even if treatment may be lifelong (Dimosiari et al. 2023).

An additional biologic, the anti-IL-6 receptor (anti-IL-6R), which is used to dampen and reduce inflammation, has been associated with minor, but manageable, CVD side effects (Chen et al. 2015). Patients on this treatment often experience a transient increase in LDL cholesterol and triglycerides (Chen et al. 2015); however, as described above, for RD patients that have abnormally low LDL and triglyceride levels as a consequence of their disease, treatment with anti-IL-6R could, in fact, prove beneficial. For RD individuals with normal/high levels of LDL or triglycerides, however, use of this biologic may warrant increased observation and/or need for co-use with statin therapies to minimize any added CVD risk (Emery et al. 2008).

## RD, CVD, and surgery

In addition to the increased risk of death consequent to CVD, women have increased risk of dying from cardiovascular surgery (Dixon et al. 2022). Disturbingly, data from nearly 1.3 million cardiac bypass surgeries show that women have a 30%–40% increased risk of death as compared to men (Gaudino et al. 2023). Unfortunately, information on the mortality and complications associated with cardiac surgery in women with RDs specifically are not fully understood. What is known, however, comes from small, single site studies with few participants, but indicate, nonetheless, that there is an increased risk of mortality in individuals with RA following coronary artery bypass surgery, likely due to preoperative use of corticosteroids (Malmberg et al. 2021). Moreover, small cohort studies further demonstrated that RD patients were at higher risk of developing postoperative complications, with one study citing up to 44% of SLE patients suffering bleeding complications post cardiovascular surgery (Lin et al. 2005). Valvular heart disease and pericarditis requiring surgical intervention in the form of valve replacement or pericardial window surgery are, unfortunately, quite frequent in SLE (Doria et al. 2005). In this regard, according to a single site study, at least 63% of SLE patients had at least one major complication following CVD surgical intervention and suffered a mortality rate of 27%, primarily due to infection (Tejeda-Maldonado et al. 2018).

It is concerning that RD patients, especially women, face this double burden of RD and increased CVD-related death risk. Indeed, the relationship between pharmacological therapeutics, complications, and mortality rates in RD need further investigation. Having an increased risk of serious infection has also been a long-known side effect, not only of surgical intervention, but also of the many immunomodulating therapeutics used to treat RD, including the biologics. To complicate matters further, there are important considerations needed for managing individuals with RDs post-surgery due to the nature of their pathophysiology, including handling complexities such as abnormal clotting and development of antiphospholipid syndrome post cardiac surgery (Yamamoto et al. 2023). In this regard, there is no consensus; how to manage pharmacological therapy to minimize deleterious and potentially fatal complications in RD, particularly those with associated CVD risk, remains elusive.

## Future directions

Involving cardiologists in RA care could reduce CVD risk and associated mortality (Guerra et al. 2022). Given that the prevalence of RDs is greater in women than men, and that the field of cardio-rheumatology is entering its formative years, an opportunity to propel our understanding of RDs and improve the care of women is now. While the association between CVD and mortality in women with RD has become clear, the underlying functional and molecular mechanisms remain yet to be elucidated. We need to do more on this front, not only to understand the pathophysiology, but to help identify, target, and develop drugs that can be more specific and efficacious for the treatment of RDs.

Long term, there are many unanswered questions on the interplay of RD pathologies and their effects on lifestyle, environmental risks, the consequences of pharmaceutical and surgical interventions, and how these relate to CVD manifestation and risk management. Making accurate risk assessments of a patient’s likelihood of developing CVD requires an understanding of the contributions of each of these risk factors. Further work on delineating the contributions of each of these areas to patient risk will provide improved guidance on pharmacological management of RD patients in a CVD context. We firmly believe that expanding cardio-rheumatology subspecialist awareness, training, and research would help significantly in improving the care of women with RDs, both in the short and long term.

## Conclusion

In general, women have an increased risk of death and complications due to CVD, despite our awareness and treatment strategies for these disorders over the past several decades. Women with an RD also suffer the compounded increased risk of mortality and complication, not only due to the RD itself, but also to the treatments for the disease as well. Holistic care of RD, inclusive of cardiac disease intervention, is critical in providing long term risk minimization, disease control, and improvements in quality of life and mortality of these patients. The current fundamental problems limiting the new subspecialty of cardio-rheumatology are the lack of information and evidence regarding sex-differences in CVD presentation and management, as well as the risk assessment of the side effects associated with CVD interventions. Therefore, there is an urgent and immediate need for large scale clinical studies to (1) identify early warning signs of CVD in women with RD to allow early intervention and to prevent the progression of CVD, (2) identify the source of increased mortality in women after cardiovascular surgery to implement care plans which mitigate risk, and (3) increase awareness of increased CVD risk in female RD patients in primary care practitioners to ensure prompt referral and access to specialist cardio-rheumatology care. Given the specialist training cardio-rheumatologists receive, they are in a unique position to answer these questions and provide new pathways forward for better management and therapeutic modalities for RD patients.

## Figures and Tables

**Table 1.** Rheumatic diseases (RDs) have a stark sex bias, indicating that this class of diseases is much more common in women than men.

Rheumatic disease	Female: male ratio
Ankylosing spondylitis	1:2
Antiphospholipid syndrome	5:1
Autoimmune chronic hepatitis	7:1
Celiac disease	1:1
Graves’ disease	7:1
Hashimoto’s disease	19:1
Multiple sclerosis	2:1
Myasthenia graves	3:1
Biliary cirrhosis	10:1
Psoriasis	1:1
Rheumatoid arthritis	3:1
Sjogren’s syndrome	9:1
Systemic lupus erythematosus	9:1
Systemic sclerosis	5:1
Type 1 diabetes	1:1
Hemolytic anemia	1:1
Vitiligo	1:1
Mixed connective tissue disease	8:1
Scleroderma	3:1
Chronic idiopathic thrombocytopenic purpura	2:1
Giant cell arteritis	2.5:1

**Note:** Table summarizing a variety of RDs and their incidence in women verses men, expressed as female: male ratio.

**Table 2.** Cardiovascular manifestations of rheumatic diseases (RDs).

Rheumatic disease	Cardiovascular manifestations
Ankylosing spondylitis	Aortic insufficiency, aortitis, atrio-ventricular branch block
Antiphospholipid syndrome	Valvular dysfunction, pulmonary hypertension, MI, intracardiac thrombi, ventricular dysfunction
Celiac disease	Atrial fibrillation
Graves’ disease	Heart rhythm disorders, heart failure
Hashimoto’s disease	Arrhythmias, cardiomegaly, hyperlipidemia, cardiomyopathy
Multiple sclerosis	Cardiomyopathy, autonomic nerve dysfunction, hyperlipidemia, accelerated atherosclerosis
Myasthenia graves	Heart failure, arrhythmia, myocarditis
Biliary cirrhosis	Autonomic nerve dysfunction
Psoriasis	Increased CVD risk
Rheumatoid arthritis	Increased CVD risk
Sjogren’s syndrome	Increased CVD risk
Systemic lupus erythematosus	Hypertension, accelerated atherosclerosis, pericarditis, endocarditis, myocarditis
Systemic sclerosis	Pulmonary congestion, heart failure, pulmonary hypertension, myocardial fibrosis, myositis, pericardial disease
Type 1 diabetes	Vascular damage due to uncontrolled blood sugars
Hemolytic anemia	Arrhythmia, cardiomyopathy, heart failure
Vitiligo	Increased CVD risk
Mixed connective tissue disease	Pericarditis, mitral valve prolapse, intimal hyperplasia of coronary arteries, myocarditis, pulmonary hypertension
Scleroderma	Arrythmia, congestive heart failure
Chronic idiopathic thrombocytopenic purpura	Thrombosis, accelerated atherosclerosis
Giant cell arteritis	Direct granulomatous attack of thoracic aorta and its major branches

**Note:** RDs can, in and of themselves, cause cardiovascular pathologies that can lead to significant co-morbidities in patients. Abbreviations: MI, myocardial infarction; CVD, cardiovascular disease.

**Table 3.** Cardiovascular side effects of common anti-rhematic drugs.

Drug	Class	Cardiovascular effects
Prednisone	Corticosteroid	Dyslipidemia, sodium and fluid retention, hypertension, thrombosis, vasculitis, metabolic disease
Ibuprofen	NSAID—nonselective COX1/COX2 inhibitor	Increased risk of MI, thrombotic events, heart failure and stroke. Can cause blood pressure elevation.
Naproxen	NSAID—COX1 inhibitor	Increased risk of MI, thrombotic events, and stroke. Can cause blood pressure elevation.
Diclofenac	NSAID—COX2 inhibitor	Increased risk of MI, thrombotic events, heart failure and stroke. Can cause blood pressure elevation.
Etoricoxib	NSAID—COX2 inhibitor	Increased risk of MI, thrombotic events, heart failure and stroke. Can cause blood pressure elevation.
Celecoxib	NSAID—COX2 inhibitor	Increased risk of MI, thrombotic events, heart failure and stroke. Can cause blood pressure elevation.
Lumiracoxib	NSAID—COX2 inhibitor	Increased risk of MI, thrombotic events, heart failure and stroke. Can cause blood pressure elevation.
Rofecoxib	NSAID—COX2 inhibitor	MI, stroke, cardiovascular death; withdrawn from market
Methotrexate	DMARD—folate antagonist	Cardioprotective
Hydroxychloroquine	DMARD—cathepsin L inhibitor	Heart rhythm abnormalities
Sulfasalazine	DMARD—dihydropteroate synthase inhibitor	Pleuropericarditis
Leflunomide	Pyrimidine synthesis inhibitor.	Hypertension
Etanercept	Biologic TNF*α* blocker	Ventricular remodeling, myocyte fibrosis
Infliximab	Biologic TNF*α* blocker	Hypertension, arrhythmia
Adalimumab	Biologic TNF*α* blocker	Hypertension, pericarditis
Certolizumab pegol	Biologic TNF*α* blocker	Worsens heart failure
Golimumab	Biologic TNF*α* blocker	Heart failure, hypertension
Abatacept	Biologic IgG1-CTLA fusion protein	Hypertension
Rituximab	Biologic anti-CD20 antibody	Arrhythmia, MI
Tocilizumab	Biologic anti-IL-6R antibody	Hypertension, transient dyslipidemia
Anakinra	Biologic IL-1R antagonist protein.	Cardioprotective
Triamcinolone	Corticosteroid	Premature atherosclerosis, hypertension, fluid retention, arrhythmia
Methylprednisolone	Corticosteroid	Premature atherosclerosis, hypertension, fluid retention, arrhythmia, hyperlipidemia
Ruxolitinib	Small molecule competitive JAK1/2 inhibitor	MI, thrombosis, heart failure

**Note:** MI, myocardial infarction; COX, cyclooxygenase; NSAID, non-steroidal anti-inflammatory drug; DMARD, disease modifying anti-rheumatic drug; TNF*α*, tumor necrosis factor alpha; IL-6R, interleukin 6 receptor; JAK, Janus kinase; IgG1, immunoglobin G 1; CTLA, cytotoxic T-lymphocyte-associated protein; IL-1R, interleukin 1 receptor; CD20, cluster of differentiation 20.

## Data Availability

This manuscript does not report data.
